# Le synovialosarcome du poumon: à propos d’un cas et revue de la littérature

**DOI:** 10.11604/pamj.2020.36.137.23034

**Published:** 2020-06-29

**Authors:** Onana Remy, Mohammed Messouna, Gloria Akimana, Marius Kamdem, Hassan Errihani

**Affiliations:** 1 Service d’Oncologie Médicale, Institut National d’Oncologie, Rabat, Maroc,; 2 Service de Chirurgie Thoracique, Hôpital Militaire d’Instruction Mohamed V, Rabat, Maroc

**Keywords:** Synovialosarcome, tissus mous, cytogénétique, Synovial sarcoma, soft tissue, cytogenetics

## Abstract

Le synovialosarcome est une tumeur maligne rare, soit 10% des sarcomes des tissus mous. Il se développe habituellement au niveau des membres et sa localisation pulmonaire est exceptionnelle. Nous rapportons un cas clinique de synovialosarcome du poumon chez un homme de 54 ans découvert à un stade localisé. Cette tumeur, extrêmement rare, présente un phénotype immunohistochimique particulier, qui contribue fortement au diagnostic. L'étude cytogénétique confirme le diagnostic en montrant la présence de la translocation spécifique t (X; 18), qui caractérise le synovialosarcome quelle que soit sa localisation anatomique. À travers cette observation, nous insistons sur les caractéristiques anatomo-cliniques, thérapeutiques et pronostiques de cette tumeur rare souvent méconnue par les cliniciens.

## Abstract (en)

Pulmonary synovial sarcoma is a rare malignant tumor accounting for 10% of soft tissue sarcomas. It usually arises from the limbs and its occurrence in the lung is exceptional. We here report a clinical case of pulmonary synovial sarcoma in a 54-year-old man detected at a localised stage. This extremely rare tumor has a specific immunohistochemical phenotype. This provides strong support for diagnosis. Cytogenetic study confirms the diagnosis by showing the presence of specific translocation t (X; 18). This is highly characteristic of synovial sarcoma regardless of its anatomical location. This study highlights the anatomo-clinical, therapeutic and prognostic characteristics of this rare tumor often unknown to clinicians.

## Introduction

Les sarcomes des tissus mous sont des tumeurs rares, caractérisées par une grande hétérogénéité anatomique, histologique et pronostique. L’incidence générale des sarcomes des tissus mous est estimée entre 3 et 4 pour 100 000 habitants et 50 à 60% de ces cancers se développent au niveau des membres [1]. Les sarcomes primitifs thoraciques sont rares et représentent moins de 1% de l’ensemble des tumeurs thoraciques primitives [2]. Le synovialosarcome est le 4^e^sarcome des tissus mous (8 à 10%) en termes de fréquence; il affecte l’adule jeune (15-40 ans) avec une légère prédominance masculine [3]. Une trentaine de cas de synovialosarcome de siège primitif pulmonaire est décrite dans la littérature. Nous rapportons une nouvelle observation en présentant les particularités de cette tumeur peu rencontrée en pratique clinique.

## Patient et observation

**Figure 1:**
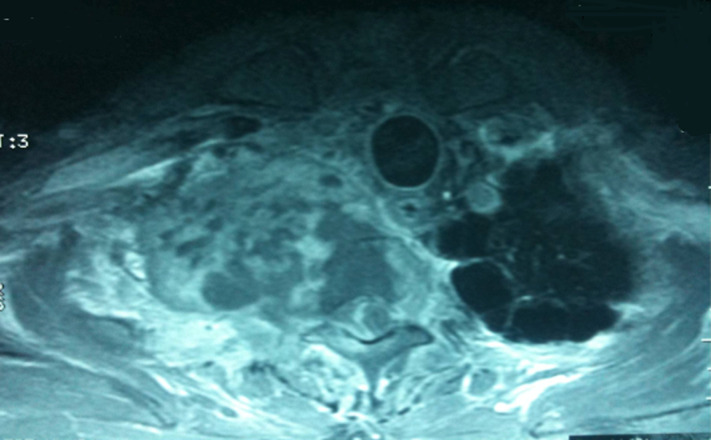
image tomodensitométrique montrant l’envahissement pulmonaire droit du synovialosarcome

Monsieur H.M est un patient âgé de 54 ans, tabagique chronique à 25 paquets-années et sevré, a consulté en mai 2017 pour une douleur thoracique et une dyspnée associées à une toux produisant des crachats hémoptoiques en contexte d’altération de l’état général. L’examen physique était sans particularités. Par ailleurs, il n’a pas été retenu une notion de contage tuberculeux. Un scanner thoraco-abdomino-pelvien a été demandé et a montré un processus tumoral pulmonaire périphérique (7,5x5x6cm) lobaire moyen et supérieur droit associé à un nodule homolatéral. L’étage abdominal ne présentait pas d’anomalie (Figure 1). Le patient a bénéficié d’une lobectomie supérieure et moyenne droite avec curage ganglionnaire le 26.07.2017.

L’examen anatomopathologique de la pièce opératoire était en faveur d’un sarcome à cellules fusiformes de grade II (synovialosarcome) classé pT3N0Mx avec des limites chirurgicales saines sans emboles vasculaires ni d’engainements péri-nerveux. Une relecture des lames a été faite en faveur du diagnostic de synovialosarcome. Le complément d’immunohistochimie a montré une positivité pour le marqueur anti-EMA et une négativité pour les marqueurs CD34, CD20, PS100 et à l’anti-cytokératine type AE1/AE3. La recherche d’un transcrit de fusion spécifique de la translocation t (X, 18) s’est révélée positive par la technique de FISH. Le patient a été mis sous surveillance et a présenté une récidive localement avancée, jugée non opérable à 6 mois post-opératoire. Une chimiothérapie palliative type doxorubicine a été administrée soit un total de 5 cures; arrêtée en janvier 2019 à cause de l’altération de l’état général et l’aggravation de la dyspnée. Le patient est décédé en avril 2019 dans un tableau de détresse respiratoire.

## Discussion

Les sarcomes des tissus mous sont des tumeurs rares, caractérisées par une grande hétérogénéité anatomique, histologique et pronostique. L’incidence des sarcomes des tissus mous est estimée entre 3 et 4 pour 100 000 habitants et 50 à 60% de ces cancers se développent au niveau des membres [1]. Les sarcomes primitifs thoraciques sont rares et représentent moins de 1% de l’ensemble des tumeurs thoraciques primitives [2]. Le synovialosarcome est le 4^e^ sarcome des tissus mous (8 à 10%) en termes de fréquence; il affecte l’adule jeune (15-40 ans) avec une légère prédominance masculine [3]. L’âge de notre patient est de 54 ans, donc de loin supérieur aux données de la littérature mais, rejoint la série de Mastroianni et al dont la moyenne d’âge des patients était comprise entre 50 et 60 ans [4]. La plupart des sarcomes n’ont pas été associés à des facteurs de risque mais, certaines prédispositions génétiques et environnementales ont été suggérées chez une minorité de patients [5].

Sur le plan clinique, les patients atteints de synovialosarcome du poumon consultent généralement pour une douleur thoracique, une toux; une hémoptysie et une dyspnée d’aggravation progressive sur plusieurs mois [6]. Le tableau de notre patient est typique de cette description. Sur le plan radiologique, on retrouve une masse contenant des calcifications dans 25% des cas. La tomodensitométrie permet de mieux apprécier la présence des micro-calcifications ainsi que l’extension endothoracique et pariétale. À l’imagerie par résonnance magnétique, environ 90% des synovialosarcomes sont bien limités avec un aspect en capsule; la présence de lobulations ou de cloisons est fréquente. Les tumeurs sont hétérogènes en T2 avec des signaux de tonalité liquidienne, solide ou fibreuse. L’utilité de la tomographie par émission de positons (TEP-TDM) a été peu étudiée et la scintigraphie osseuse est préconisée en cas de point d’appel osseux [6].

**Figure 2:**
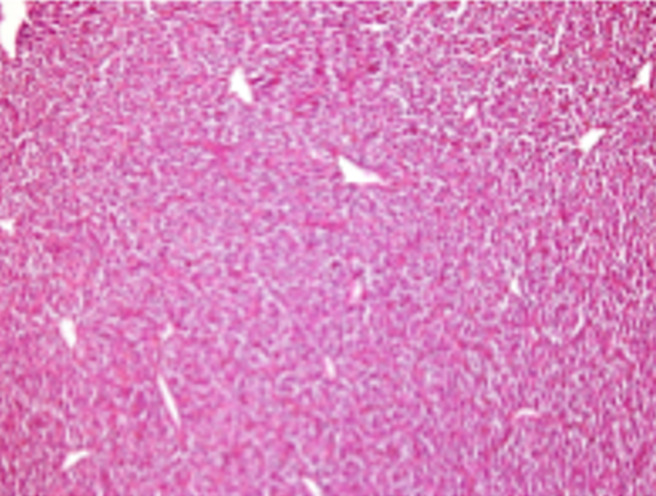
examen morphologique du synovialosarcome monophasique (cellules fusiformes): examen microscopique de la tumeur (HES, grossissement x40)

Sur le plan histologique devant une prolifération tumorale maligne d’aspect sarcomateux a fortiori à cellules fusiformes (Figure 2), il convient toujours de rechercher une composante carcinomateuse afin d’éliminer le diagnostic de carcinosarcome. Une fois le caractère pur de la prolifération sarcomateuse établi, il faut écarter de prime abord l’éventualité d’un carcinome sarcomatoide. Contrairement au synovialosarcome, celui-ci est toujours riche en atypies cyto-nucléaires. De plus les cellules proliférantes y sont intensément et diffusément positives aux marqueurs épithéliaux. Après avoir écarté ces 2 éventualités plus fréquentes le diagnostic de sarcome peut être retenu. Se pose alors la question s’agit-il d’une tumeur pulmonaire primitive ou secondaire? La 2^e^ éventualité est de loin la plus fréquente. La tumeur mère siège généralement dans les tissus mous. Seule l’absence de localisation tumorale extra-pulmonaire dans le passé, au moment du diagnostic et après 2 ans de recul attestera du caractère primitif de la tumeur pulmonaire. Les métastases pulmonaires concomitantes peuvent aussi être révélatrices d’un sarcome extra-thoracique [7]. Au moment du décès, notre patient ne présentait pas de localisation tumorale extra-thoracique soit environ 2 ans de recul.

On distingue trois sous-types de synovialosarcomes: la forme monophasique (31%) qui est une forme fibrosarcomateuse pure, la forme biphasique qui associe des cellules épithéliales et des cellules fusiformes (36%) et la forme peu différenciée (36%) qui contient des cellules de petite taille de forme ovalaire caractérisées par un cytoplasme peu abondant et un noyau dense. À l’immunohistochimie, les synovialosarcomes expriment dans 90% des cas, l’Epithelial Membrane Antigen (EMA) et les cytokératines, dans 60% des cas le CD99 et dans 30% des cas la protéine S100 [8]. Dans la plupart des cas, on retrouve une translocation caractéristique t (X; 18) qui implique les gènes SSX1 ou SSX2 du chromosome X (Xp11). Les transcrits du gène de fusion SYST-SSX peuvent être détectés sur des prélèvements anatomopathologiques avec une sensibilité de 96% et une spécificité de 100% [9]. Dans le cadre de notre observation, l’immunohistochimie a révélé une positivité aux anti-EMA et une négativité aux anti-cytokératines. La recherche du transcrit de fusion t (X, 18) est revenue positive par la technique d’hybridation in situ. Le score histopronostique de la Fédération Nationale des Centres de Lutte contre le Cancer (FNCLCC), qui intègre 3 paramètres histologiques: le degré de nécrose tumorale, le degré de différenciation et le pourcentage de mitoses [10]. Ce score, applicable à la grande majorité des histologies de sarcome des tissus mous, constitue un facteur pronostique puissant et facilement reproductible, permettant de séparer les sarcomes en tumeurs de bas grade (grade I), de grade intermédiaire (grade II) et de haut grade (grade III) [10].

En l’absence de métastases, la chirurgie reste le traitement de choix et une résection large est impérative pour réduire les risques de récidives loco-régionales et à distance l’intérêt de la radiothérapie adjuvante est de permettre un meilleur contrôle local de la tumeur [11]. Elle est indiquée lorsque la tumeur a un diamètre supérieur ou égal à 5cm et de marges incomplètes. Aucune étude n’a permis d’évaluer le bénéfice de la chimiothérapie adjuvante dans cette situation [11]. Un traitement par Doxorubicine et ou Ifosfamide constitue le traitement de première ligne dans les formes inopérables et dans les formes métastatiques avec un taux de réponse de l’ordre de 50% [11]. Le taux moyen de récidives loco-régionales ou métastatique à deux ans est de 50% [12]. Les sites métastatiques les plus fréquents sont ganglionnaires, osseux et hépatiques. Un diamètre tumoral inférieur à 5cm, un index mitotique faible (Ki 67 < 10%), l’absence de nécrose tumorale, l’absence de tumeur résiduelle après résection chirurgicale sont considérées comme des facteurs de bon pronostic. La survie à 5 ans varie entre 35% et 76% selon l’absence ou la présence des facteurs de bon pronostic respectivement [12]. Le rôle du transcrit de fusion comme facteur pronostic n’est pas définitivement établi [12]. Dans notre observation, le patient avait une taille tumorale à 7,5cm et un grade II. La taille tumorale et l’absence de radiothérapie adjuvante ont contribué à la récidive précoce et à l’évolution défavorable de sa maladie.

## Conclusion

Le synovialosarcome du poumon est une tumeur maligne rare. Son diagnostic est difficile et requiert une analyse immunohistochimique et cytogénétique qui permettent de le distinguer des autres tumeurs mésenchymateuses. La recherche d’un transcrit de fusion SYST-SSX est systématique et permet d’affirmer le diagnostic dans la plupart des cas. Il est important d’observer deux ans d’évolution sans atteinte extra-thoracique pour confirmer le caractère primitif pulmonaire du synovialosarcome. La chirurgie reste le traitement de référence suivie plus ou moins de la radiothérapie. Les récidives sont fréquentes et le pronostic est réservé au stade avancé avec un bénéfice modeste de la chimiothérapie. Notre cas témoigne de l’intérêt de la collaboration multidisciplinaire dans la prise en charge de cette tumeur.
